# Arthroscopically Assisted Retrograde Intramedullary Nailing for Periprosthetic Fracture of the Femur after Posterior-Stabilized Total Knee Arthroplasty

**DOI:** 10.1155/2018/1805145

**Published:** 2018-04-03

**Authors:** Kazuhiko Udagawa, Yasuo Niki, Kengo Harato, Shu Kobayashi, So Nomoto

**Affiliations:** ^1^Department of Orthopaedic Surgery, Keio University School of Medicine, Tokyo, Japan; ^2^Department of Orthopaedic Surgery, Yokohama-City Tobu Hospital, Yokohama, Japan

## Abstract

Retrograde intramedullary nailing (RIMN) has been used for periprosthetic fracture of the distal femur after total knee arthroplasty (TKA), yielding good fracture union rates and satisfactory outcomes. However, RIMN for posterior-stabilized- (PS-) TKA risks malpositioning the entry point and disturbing the post of the tibial insert, and the surgeon therefore usually requires knee joint arthrotomy. We report a case of a 79-year-old male who was involved in bicycle accident resulting in periprosthetic fracture of the distal femur after PS-TKA. We performed osteosynthesis with arthroscopically assisted RIMN to define an appropriate entry point. RIMN for posterior-stabilized- (PS-) TKA risks malpositioning the entry point and disturbing the post of the tibial insert. Because arthroscopy can directly visualize the entry point and the tibial post without arthrotomy, arthroscopically assisted RIMN offers a useful technical option for periprosthetic fracture of the distal femur after PS-TKA.

## 1. Introduction

Total knee arthroplasty (TKA) is the gold standard for patients with end-stage knee osteoarthritis and can be helpful to correct deformity, relieve pain, and restore function. With the shift toward an increasingly aging society, the number of TKAs is expected to increase. Incidences of primary and revision TKAs have been reported as 0.6% and 1.7%, respectively [1], suggesting that post-TKA periprosthetic fracture is also increasing. Open reduction and internal fixation with a nonlocked plate or condylar plate has been the standard for post-TKA periprosthetic fracture, but clinical results have been poor, with complication rates up to 53% [2]. For now, both retrograde intramedullary nailing (RIMN) and use of locking compression plates (LCPs) have become common for the treatment of the periprosthetic fracture after TKA, and these techniques have achieved improved union rates and functional outcomes [3]. Although a recent meta-analysis demonstrated similar clinical outcomes between RIMN and the use of LCPs [3], RIMN cannot be indicated for certain designs of the femoral component, such as a stemmed design, posterior-stabilized- (PS-) TKA and closed box design. In addition, the post of the polyethylene insert is at risk of breakage during reaming, regardless of the PS-TKA design. Even for cruciate-retaining- (CR-) TKA or open box PS-TKA, the entry point for RIMN cannot be detected accurately even using fluoroimages, due to overlap with both femoral condyles. Consequently, surgeons inevitably perform knee joint arthrotomy.

Currently, we use arthroscopy to accurately detect the appropriate entry point for RIMN intraoperatively. In this article, we present an arthroscopically assisted RIMN technique for the treatment of periprosthetic fracture after closed box PS-TKA.

## 2. Case Presentation

**Figure 1:**
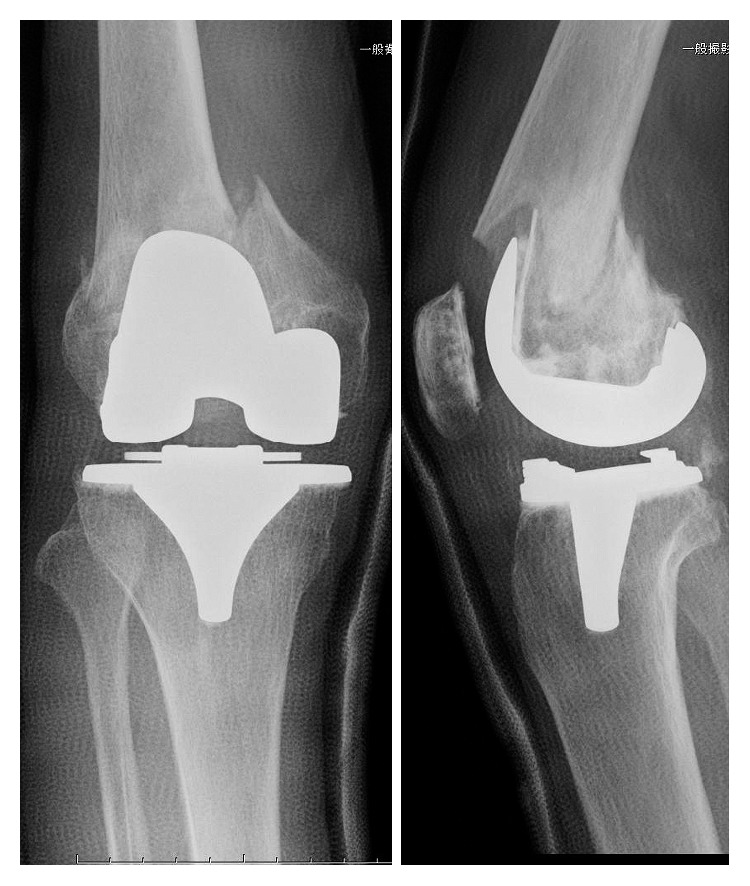
Preoperative radiograph.

A 79-year-old man with right knee osteoarthritis underwent PS-TKA (Vanguard System; Zimmer Biomet, Tokyo, Japan). At 10 months postoperatively, he was involved in a bicycle accident and visited a local hospital, where he was diagnosed with periprosthetic fracture of the distal femur (Figure 1).

Three days after the accident, he was referred to our hospital, and osteosynthesis with arthroscopically assisted RIMN was performed.

**Figure 2:**
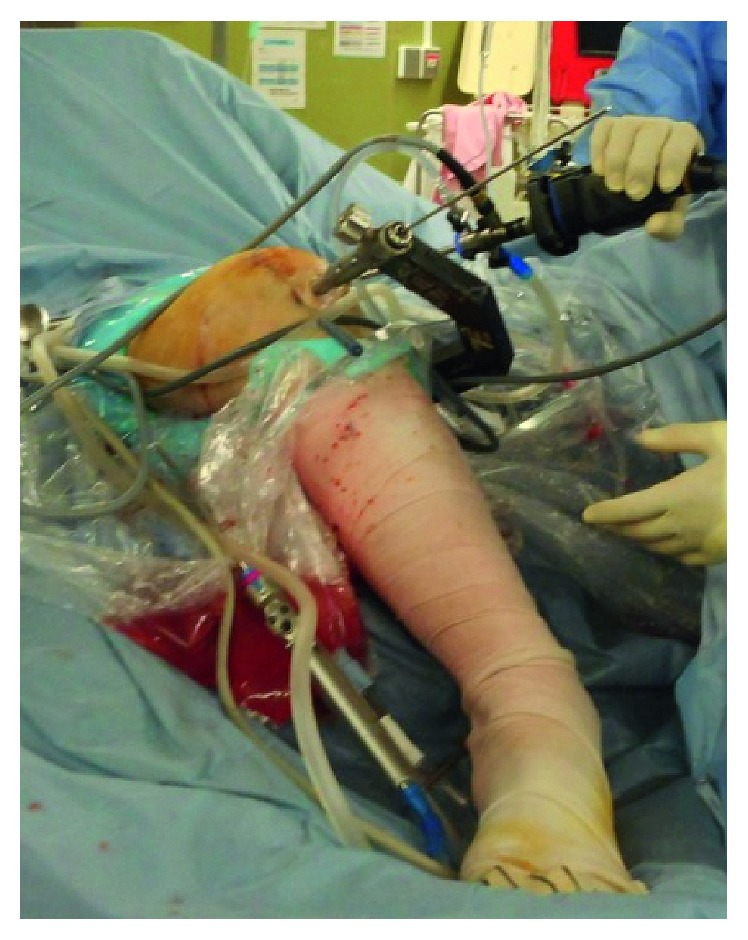
Knee joint positioning and set up of image intensifier.

The patient was positioned supine with a standard leg holder, and the knee was flexed to 90° to allow the nail to pass behind the femoral shield on a radiolucent fracture table (Figure 2).

**Figure 3:**
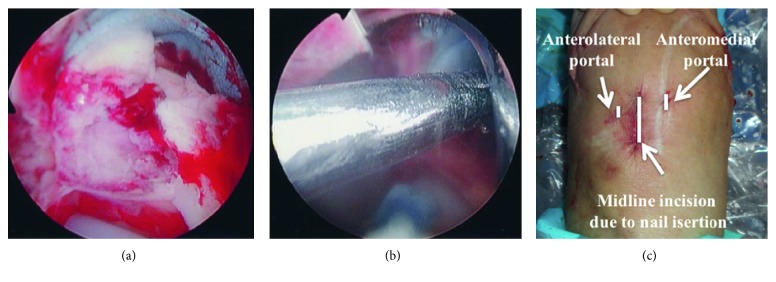
Synovectomy and exposure of insertion point (a). Guide-wire insertion (b). Skin incisions for arthroscopy and nail insertion (c).

Synovectomy was performed under arthroscopy, and the entry point between the condyles of the femoral component was identified using a standard anterolateral portal and an anteromedial portal (Figure 3(a)). 3 cm midline incision was made and the patellar tendon was split, and then the guide-wire was inserted (Figures 3(b) and 3(c)).

**Figure 4:**
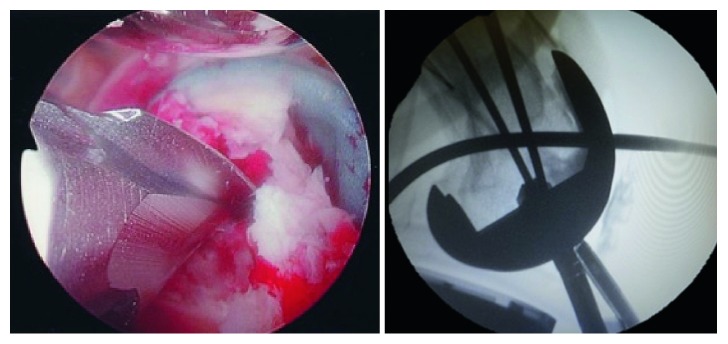
Entry point reaming without compromising the tibial post.

After definitive guide-wire placement, the entry point was reamed without compromising the tibial post, and a ball-tip guide rod was inserted into the canal of the proximal femur (Figure 4).

**Figure 5:**
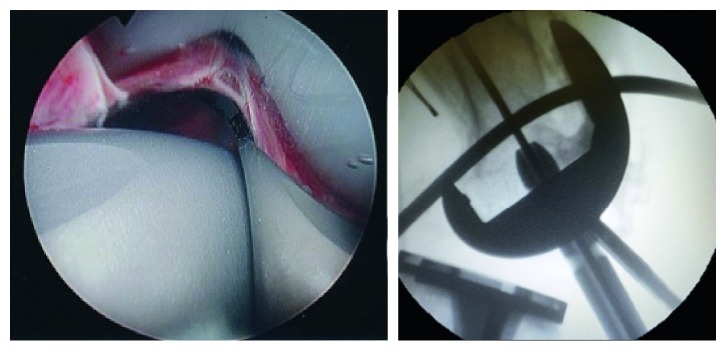
Nail insertion.

As the distal fragment was shifted posteriorly, a 3.0 mm Kirschner wire was inserted just posteriorly from the ball-tip guide rod in a proximal fragment as a block pin as reported previously [4]. The intramedullary canal was reamed, and a 12 mm diameter × 170 mm length T2 Supracondylar Nail (Stryker, Schönkirchen, Germany) was then inserted (Figure 5).

**Figure 6:**
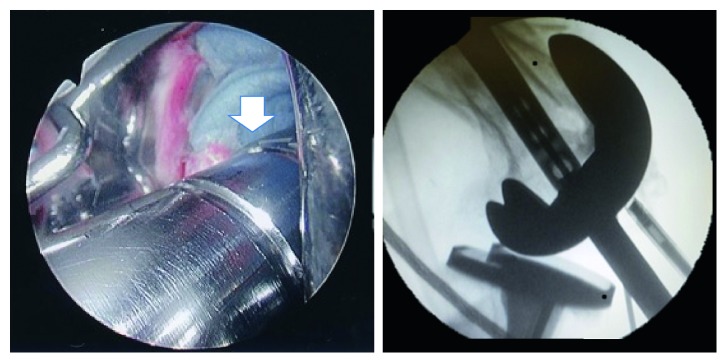
White arrow indicates the end of the nail.

To acquire the appropriate positioning of the distal locked screws and prevent the end of the nail from compromising the tibial post after surgery, the depth of the nail should be placed just at the end of the distal femur (Figure 6).

**Figure 7:**
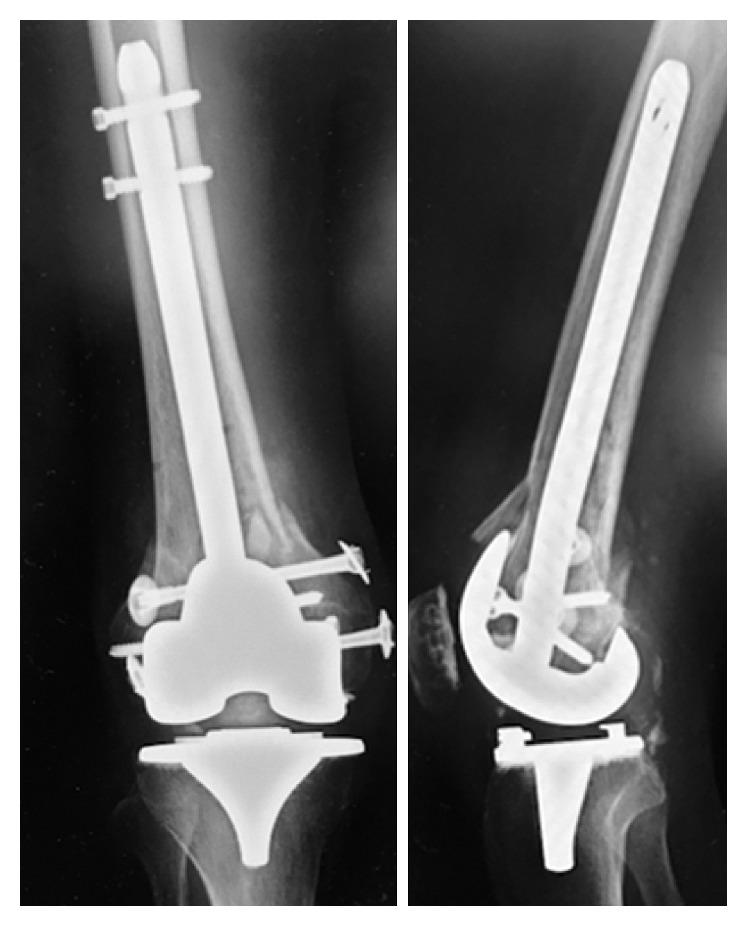
Postoperative radiograph.

The proximal and distal locked screws were then inserted, followed by removal of the jig. Finally, nail impingement on the tibial post was confirmed using arthroscopy, and the patellar tendon was repaired. Postoperative radiograph indicated good sagittal and coronal alignment of the distal femur (Figure 7).

He exhibited good recovery with knee range of movement from 5° to 120° at 4 weeks postoperatively and successfully returned to preinjury functional activities at 4 months.

## 3. Discussion

Various clinical characteristics are encountered with periprosthetic fracture after TKA, such as poor bone quality, delayed fracture healing, and prosthetic loosening. Fracture without displacement around a stable prosthesis may be treated conservatively, while internal fixation is the treatment option for displaced periprosthetic fracture without component loosening. Although the current findings suggested that both RIMN and use of an LCP could be indicated for these fractures [3], the priority of the implant choice remains controversial. The LCP may be applied in nearly all periprosthetic fracture situations without loosening. However, some drawbacks have been identified in the use of LCP. Potential periosteal stripping may violate the periosteal blood supply and interrupt bony union. Hoffmann et al. reported that the nonunion rate reached 22.2%, and hardware failure was observed in 8.3% of their LCP series of 55 consecutive periprosthetic fractures after TKA [5]. Moreover, Johnston et al. reported that an LCP caused irritation of the iliotibial band and soft-tissues of the knee, leading to premature removal in a separate anesthetic session [6].

RIMN is less invasive than the LCP technique, due to greater sparing of the fracture site, with minimal soft-tissue dissection and avoidance of periosteal stripping. However, some authors have advocated postoperative malalignment of RIMN. Lee et al. reported that 16% of procedures developed malalignment, including hyperextension and valgus alignment [7] according to Rorabeck and Taylor criteria [8]. To avoid hyperextension of the femoral component and damage to the tibial post during reaming, accurate identification of the appropriate entry point and direction of nail insertion are paramount. Because the entry point overlaps with the femoral component even under intraoperative fluoroimaging, accurate detection of the entry point and tip of the tibial post intraoperatively is difficult. With the technique we have described here, arthroscopy can directory visualize the entry point and the tibial post.

In cases with a small distal fragment, in order to insert multiple distal locked screws in RIMN, the distal end of the nail should be placed just at the surface of the intercondylar notch. Using arthroscopy, the end of the nail can be positioned as distally as possible, and RIMN is thus indicated for distal periprosthetic fractures with a small distal fragment.

Several contraindications for the present technique should be taken into account. Our technique is not indicated for patients with a stiff knee. If preoperative flexion angle is less than 90°, we cannot insert the nail using arthroscopy. In cases with the femoral component placed in flexion, the appropriate position of the entry point would be obscured. The diameter of medullary canal sometimes becomes a point of discussion. When the canal diameter of the isthmus is smaller than the smallest diameter of retrograde nails, the nail cannot be inserted through the canal of the femur. Thus, the surgeons should pay careful attention to whether the intramedullary canal diameter matches the nail diameter before surgery. Moreover, compatibility of the retrograde nail and TKA prostheses is a more critical issue. Most standard-sized retrograde nails are able to be technically inserted through most TKA prostheses. However, the recent study using sawbones indicated that six of the eight commonly used TKA prostheses scratched the nail or excessive force was needed on insertion [9]. From this perspective, our arthroscopically assisted technique can monitor and prevent the contact between the nail and the prosthesis during insertion.

In conclusion, this arthroscopically assisted technique is less invasive and useful for periprosthetic fracture when considering RIMN for periprosthetic fracture of the distal femur after PS-TKA.

## References

[1] Meek R. M., Norwood T., Smith R., Brenkel I. J., Howie C. R. (2011). The risk of peri-prosthetic fracture after primary and revision total hip and knee replacement. *Journal of Bone and Joint Surgery*.

[2] Herrera D. A., Kregor P. J., Cole P. A., Levy B. A., Jonsson A., Zlowodzki M. (2008). Treatment of acute distal femur fractures above a total knee arthroplasty: Systematic review of 415 cases (1981–2006). *Acta Orthopaedica*.

[3] Shin Y. S., Kim H. J., Lee D. H. (2017). Similar outcomes of locking compression plating and retrograde intramedullary nailing for periprosthetic supracondylar femoral fractures following total knee arthroplasty: a meta-analysis. *Knee Surgery, Sports Traumatology, Arthroscopy*.

[4] Krettek C., Miclau T., Schandelmaier P., Stephan C., Mohlmann U., Tscherne H. (1999). The mechanical effect of blocking screws (“Poller screws”) in stabilizing tibia fractures with short proximal or distal fragments after insertion of small-diameter intramedullary nails. *Journal of Orthopaedic Trauma*.

[5] Hoffmann M. F., Jones C. B., Sietsema D. L., Koenig S. J., Tornetta P. (2012). Outcome of periprosthetic distal femoral fractures following knee arthroplasty. *Injury*.

[6] Johnston A. T., Tsiridis E., Eyres K. S., Toms A. D. (2012). Periprosthetic fractures in the distal femur following total knee replacement: a review and guide to management. *Knee*.

[7] Lee S. S., Lim S. J., Moon Y. W., Seo J. G. (2014). Outcomes of long retrograde intramedullary nailing for periprosthetic supracondylar femoral fractures following total knee arthroplasty. *Archives of Orthopaedic and Trauma Surgery*.

[8] Rorabeck C. H., Taylor J. W. (1999). Classification of periprosthetic fractures complicating total knee arthroplasty. *Orthopedic Clinics of North America*.

[9] Jones M. D., Carpenter C., Mitchell S. R., Whitehouse M., Mehendale S. (2016). Retrograde femoral nailing of periprosthetic fractures around total knee replacements. *Injury*.

